# The complete mitochondrial genome of *Tylototriton anhuiensis* and implications for its taxonomy

**DOI:** 10.1080/23802359.2018.1535843

**Published:** 2018-10-26

**Authors:** Xue Han, Guiyou Wu, Lifu Qian, Xiaonan Sun, Baowei Zhang, Tao Pan

**Affiliations:** a College of Nature Resource and Environment, Chizhou University, Chizhou, Anhui, China;; b School of Life Sciences, Anhui University, Hefei, Anhui, China;; c School of Life Sciences, Anhui Normal University, Wuhu, Anhui, China

**Keywords:** *Tylototriton anhuiensis*, mitogenome, phylogentic tree

## Abstract

*Tylototriton anhuiensis* (Salamandridae, Urodela), collected from Yaoluoping Nature Reserve, was identified as a new species. The complete mitogenome sequence of *T. anhuiensis* is 16,259 by in length, including 13 protein-coding genes, 22 tRNA genes, 2 rRNA genes, and D-loop region. The base composition of the mitogenome was 33.6%A, 26.3% C, 14.5% G, and 25.6% T. The *ND6* subunit gene and eight tRNA genes were encoded on the L-strand, the others were encoded on the H-strand.

*Tylototriton anhuiensis* (Salamandridae, Urodela) was identified as a new species based on these specimens collected from Yaoluoping Nature Reserve (N 30°57'57.06", E 116°04'04.96") in the Dabie Mountains, Anhui, China (Qian et al. 2017), which can be distinguished from *T. wenxianensis*, *T. broadoridgus*, and *T. dabienicus* by several morphological characters, including head length is greater than width of head, bony ridges on the head are notable and necked-in, and distal digit ends, ventral digits, peripheral area of cloaca and tail lower margin are orange.

The specimen used in our study is stored in the museum of Anhui University (AHU) in Heifei, Anhui, China. We chose the muscle tissue to extract the whole genomic DNA using a standard proteinase-k/phenol-chloroform protocol (Sambrook and Russell 2006**).** The entire mitogenome was amplified using 15 pairs of primers by PCR. Here, we sequenced the complete mitochondrial genome of *T. anhuiensis* sp.nov. Phylogenetic relationship of seven *Tylototriton* species from GenBank, *T. anhuiensis* and *T. kweichowensis* in research were analyzed with the Bayesian inference (BI) analyses using the MrBayes v.3.2.2 software **(**Huelsenbeck and Ronquist 2001**)**, selecting *Pleurodeles waltl*, *Echinotriton andersoni* as outgroups. In this process, the best-fitting nucleotide substitution model (GTR + I + G) was selected via MrModeltest version 2.1 (Nylander et al. 2004); the four independent Markov’s chain runs for 1,000,000 metropolis-coupled Monte Carlo (MCMC) generations, sampling every 1000 generations. The first 1000 trees were discarded as ‘burn-in’. The maximum likelihood (ML) analyses were performed in RAxML v.8 (Stamatakis 2014) with a general time reversible model of nucleotide substitution under the Gamma model of rate heterogeneity (GTRCAT). Support for the internal branches was evaluated with 1000 bootstrap replicates.

The complete mitogenome sequence of *T. anhuiensis* is 16,259bp in length, including 13 protein-coding genes, 22 tRNA genes, 2 rRNA genes, and a *D-loop* region. The base composition of the mitogenome was 33.6%A, 26.3% C, 14.5% G, and 25.6% T. The *ND6* subunit gene, and eight tRNA gene were encoded on the L-strand, the others were encoded on the H-strand. In 13 protein-coding genes, the shortest one is *ATP8* gene (168 bp) and the longest one is the *ND5* gene (1811 bp). For most protein-coding genes, they take ATG as start codons, while *COX I* regard GTG as start codons. The D-loop of the *T. anhuiensis* mtDNA was 738 bp in length.

According to the Bayesian analysis, the phylogenetic tree divided into two well-supported clades (Figure 1), which correspond to two subgenus (*Tylototriton* and *Yaotriton*). The *Tylototriton* clade contains five species (*T. shanjing, T. yangi, T. verrucosus, T. kweichowensis T. taliangensis*), the *Yaotriton* clade is composed of *T. wenxianensis, T. anuiensis, T. asperrimus.* The phylogenetic result pointed out that the relationship of *T. asperrimus*, *T. wenxaanensas*, and *T. anhuiensis* was very close to each other, which explained the change in the process of species name during the three species. Our study of *T. anhuiensis* can provide a useful database for analyzing the classification and status in *Tylototriton*.

**Figure 1.**
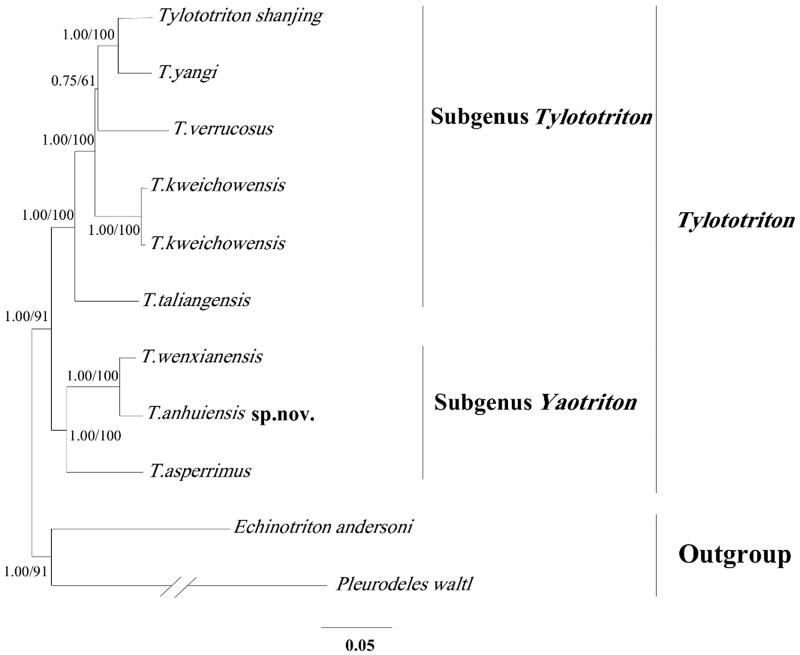
The Bayesian tree and maximum-likelihood (ML) tree among eight *Tylototriton* species based on the whole mitogenome. Numbers at the nodes are bootstrap values of the Bayesian and ML analysis. The GenBank accession number of species are *Tylototriton shanjing* (NC027505), *T. yangi* (NC032308), *T. verrucosus* (NC017871), *T. kweichowensis* (NC029231), *T. taliangensis*(NC027421), *T. wenxianensis* (NC027507) *T. asperrimus* (EU880340), *Echinotrion andersoni* (EU880314) and *Pleurodeles waltl* (EU880330).
